# Anodal transcranial direct current stimulation (tDCS) over the left dorsolateral prefrontal cortex improves attentional control in chronically stressed adults

**DOI:** 10.3389/fnins.2023.1182728

**Published:** 2023-06-15

**Authors:** Yong Liu, Qingjin Liu, Jia Zhao, Xuechen Leng, Jinfeng Han, Feng Xia, Yazhi Pang, Hong Chen

**Affiliations:** ^1^Key Laboratory of Cognition and Personality (Ministry of Education), Southwest University, Chongqing, China; ^2^School of Psychology, Southwest University, Chongqing, China; ^3^The Clinical Hospital of Chengdu Brain Science Institute, School of Life Science and Technology, University of Electronic Science and Technology of China, Chengdu, China; ^4^MOE Key Lab for Neuroinformation, High-Field Magnetic Resonance Brain Imaging Key Laboratory of Sichuan Province, University of Electronic Science and Technology of China, Chengdu, China; ^5^Department of Hepatobiliary Surgery, Southwest Hospital, Army Medical University, Chongqing, China; ^6^Faculty of Psychology, Research Center of Psychology and Social Development, Southwest University, Chongqing, China

**Keywords:** chronic stress, tDCS, left DLPFC, attentional control, N2, P3

## Abstract

**Introduction:**

Chronic stress is a long-term condition that negatively affects cognitive ability and mental health. Individuals who experience chronic stress show poor attentional control. Transcranial direct current stimulation (tDCS) to the dorsolateral prefrontal cortex (DLPFC) modulates executive function domains. Therefore, it is beneficial to investigate whether tDCS of the DLPFC could improve attentional control and relieve stress in chronically stressed individuals.

**Methods:**

We assess the event-related potentials (ERPs) associated with attentional control in individuals with chronic stress after the tDCS intervention. Forty individuals were randomly assigned to either the anodal tDCS group, which received 5 sessions of the 20 min tDCS over the DLPFC (2 mA; *n* = 20), or the sham tDCS (*n* = 20). Participants’ stress levels, anxiety, depressive symptoms, and state affects were assessed and compared before and after the intervention. The ERP was collected through electroencephalography (EEG) technology during an attentional network test.

**Results:**

After the anodal tDCS, we found a significant decrease in the perceived stress scale (PSS) scores (from an average score of 35.05 to 27.75), *p* = 0.01 as well as the State-Trait Anxiety Inventory (STAI) scores, *p* = 0.002. Better performance in the attentional network test, a significant reduction in the N2 amplitudes, and an enhancement in the P3 amplitudes (both cues and targets) were also found in the anodal tDCS group.

**Discussion:**

Our study findings suggest that tDCS to the left DLPFC could effectively relieve chronic stress, potentially reflected by increased attentional control.

## 1. Introduction

According to the American Psychological Association (2014), chronic stress is caused by the “stimulation lasting for a period of time, which may lead to mental and physical weakness.” Generally, chronic stress could result from exposure to stressors for over 30 days, or to stressors that leave lasting and fixed effects on individuals for more than 30 days (Stoney et al., 1999). It is a state of continuous arousal, in which individuals perceived needs that are greater than their internal and external resources’ capacity to deal with (Gerrig, 2007). Chronic stress is considered a long-term condition that potentially impacts individuals’ mental health and cognitive function (Koenen et al., 2017). Individuals experiencing multiple psychological stressors due to chronic stress may have a series of health issues, including anxiety, insomnia, chronic pain, hypertension, and impairments in the immune system (Pruett, 2003; Lindfors et al., 2017; Jackowska et al., 2018; Zhang et al., 2018), which could ultimately result in the onset of life-threatening diseases, such as heart diseases and depression (McEwen and Gianaros, 2010). Therefore, chronic stress have prominent adverse effects on an individual’s overall health (Milner et al., 2017). In addition, the long-term persistence of stressors could impair cognitive functions (St Jacques et al., 2013; van Rooij et al., 2015). In the field of cognitive psychology, the topic of attention control (AC) is crucial and is commonly measured by the attention network test (ANT) (Fan et al., 2002). Adequate attention control ensures that among a swamp of incoming information, the relevant and correct ones get selected and sustained in a focal manner, and the potentially distracting and salient information is ignored. It is especially valuable in tasks that require the override of prepotent and automatic reactions in favor of the completion of goal-relevant actions (Shipstead et al., 2015). Attention control also reflects the ability to consciously sustain, focus and activate an individual’s attention to memory representations when encountering irrelevant and conflicting information (Shipstead et al., 2015). Electroencephalography (EEG) is a widely used tool in the investigation of the neural mechanisms underlying tDCS effects. Event-related potentials (ERPs) are a type of EEG measure that reflect the neural markers in reaction to specific visual stimuli and provide high temporal resolution, which is commonly used in research on attention control (Bradley and Keil, 2012; Hillyard, 2017). Previous studies have indicated that ERP correlates are related to different attentional networks and attentional processing stages (Williams et al., 2016; Gonçalves et al., 2018; Wieser and Keil, 2020). In a study where attention control was assessed with ANT, Williams et al. (2016) found an increase in N2 amplitudes for incongruent target conditions, which indicated that participants allocated more attentional resources to incongruent targets than to congruent targets. In a study investigating cognition fatigue through a sustained attention task, N1 component is linked to early stages of selective attention to task stimuli. More specifically, it is known to reflect early attention allocation facilitating later perceptual processing and classification of stimuli. The P3 component is related to the later stages of conscious stimulus evaluation and differentiation. Moreover, the P3 seems to also represent a reflective process of awareness that is characterized by various higher-level post perceptual processes. With the increased time spent on the sustained attention task, both N1 and P3 exhibited an increase in amplitudes (Patel and Azzam, 2005; Polich, 2007; Railo et al., 2011; Haubert et al., 2018).

Transcranial Direct Current Stimulation (tDCS) has emerged as a promising non-invasive brain stimulation technique that can modulate brain activity and improve cognitive functioning (David et al., 2018). Several studies have investigated the effects of tDCS on stress as well as stress-related disorders, such as PTSD and anxiety disorder. A past study discovered that tDCS administered to the left-side DLPFC reduced the reported negative effects of daily stressors (Austin et al., 2016). In addition, a study by Saunders et al. (2015) investigated the effects of tDCS over the left DLPFC on individuals suffering from post-traumatic stress disorder. The authors found that tDCS shifted the P3 amplitudes, typically abnormal in individuals with PTSD, toward database norms. In addition, participants also exhibited an increase in alpha peak frequency (APF) after the stimulation, which is associated with improvements in working memory (Richard Clark et al., 2004). These findings suggest that tDCS over the left DLPFC may improve cognitive impairments in individuals who are diagnosed with PTSD (Saunders et al., 2015).

Another study by Nishida et al. (2021) investigated the effects of tDCS over the left DLPFC on STAI-state anxiety and resting EEG in healthy individuals. The authors found that participants’ state anxiety decreased significantly 1 week after the tDCS. Moreover, the changes in anxiety score were correlated with the changes in the rostral anterior cingulate cortex (rACC) alpha activity, which is considered a key hub associated with anxiety and depression (Marks and Nesse, 1994; Nishida et al., 2021).

One of the areas that have received considerable attention is the application of tDCS to enhance attentional control (Mendes et al., 2022; Nejati et al., 2022; Chen et al., 2023). Chronic stress can lead to cognitive impairments, including attentional deficits. More specifically, compared with the non-stress group, the high-stress group exhibited significant deficits in attention and memory (Lupien et al., 1998). Previous studies have investigated the effects of tDCS on attentional control using ERP as electrophysiological markers. Moezzi et al. (2021) found that tDCS over the left Dorsolateral Prefrontal Cortex (DLPFC) for five consecutive days improved attentional control in healthy individuals, as measured by an increase in P600 amplitudes and a decrease in reaction time in an Integrated Visual and Auditory-Adult Edition (IVA-AE) Task (Moezzi et al., 2021). A study by Rêgo et al. (2022) found similar results. tDCS over the left DLPFC improved sustained attention in healthy individuals. The results showed that tDCS over the left DLPFC enhanced reaction and P2 amplitudes in a visual flanker task, indicating an improvement in sustained attention after the stimulation (Rêgo et al., 2022). Moreover, tDCS has been shown to have beneficial effects on stress-related cognitive impairments. The dorsolateral prefrontal cortex (DLPFC) is the core cognitive control related brain region, making it an ideal substrate for improving cognitive conflict control (Miller and Cohen, 2001; Carter and van Veen, 2007; Hare et al., 2009; Boisseau et al., 2012; Bari and Robbins, 2013). Previous studies have also revealed that tDCS administered to the DLPFC modulates individuals’ executive function (Sarkis et al., 2014; Dubreuil-Vall et al., 2019). Even a single session of anodal tDCS was sufficient enough to induce a slight, yet significant, improvement in executive function when administrated to the prefrontal cortex (PFC) (Coffman et al., 2012; Nelson et al., 2014). In addition, 10 sessions of anodal tDCS were able to induce a long-term boost in task performances, which lasted up to 4 weeks after the stimulation (Martin et al., 2013). Dubreuil-Vall et al. (2019) also concluded that the anodal tDCS administered to the left DLPFC led to a significant improvement in cognitive control, reflected by the enhanced P3 amplitudes and the decreased N2 amplitudes. They also investigated the effects of tDCS on conflict control in Attention-Deficit/Hyperactivity-Disorder (ADHD) patients. Targeting the bilateral DLPFC, the tDCS improved patients’ conflict control during a Flanker task, which was reflected by a significantly greater P3 amplitude compared with the sham group in incongruent trials. However, although both left-side and right-side stimulation induced a significant increase in P3 amplitudes, only the left-side stimulation induced significant improvements in the behavior indexes. Therefore, the right-side stimulation was not adequate enough to elicit a physiological result that could lead to any significant behavioral changes, which indicated the left DLPFC has a bigger role in modulating conflict control (Dubreuil-Vall et al., 2019).

Based on the above findings, the present study aimed to investigate the effects of repeated DLPFC tDCS on attentional control in chronically stressed individuals. The neural markers of focus were the N2 and P3 components. We hypothesized that: (1) participants’ self-reported stress, anxiety, depression, and emotions would be alleviated after the anodal tDCS intervention; (2) the reaction time during the attention task would decrease after the anodal tDCS. More specifically, the alerting, orienting, and executive control effects would improve after the stimulation; (3) the anodal tDCS group would exhibit enhanced attentional control, which would be reflected by the decreased N2 amplitudes and the increased P3 amplitude.

## 2. Materials and methods

### 2.1. Participants

The present study recruited a total of 135 students from Southwest University who were about to take the postgraduate entrance examination in China. We used the perceived stress scale (PSS) and Student Life Stress Inventory (SLSI) to select participants. Participants who scored above 28 on the PSS and scored one standard deviation above the mean on the SLSI were included in our study (Culhane et al., 2001; Sabih et al., 2013; Liu et al., 2020). In total, 40 participants (20 females, 20 males; age range: 18–26 years, M_*age*_ = 21.2 years, SD_*age*_ = 1.65) were included in our study. We randomly assigned them to either the anodal tDCS group (*n* = 20; 9 females) or the sham group (*n* = 20; 11 females). We used GPower 3.1 for *post hoc* power analyses, and the results showed that when the effect size is set to 0.2, our sample size (*n* = 40) holds a power (1- β) of 0.85. We screened all participants for individuals with normal or corrected-to-normal vision, those who are right-handed, and who have no history of severe psychological disorders. All participants were requested to refrain from consuming any kind of substances or taking medications that may potentially affect their focus for a week leading up to the experiment. All written consent were signed before participating in the study. This study was approved by the Southwest University Ethics Committee.

### 2.2. Measures

#### 2.2.1. Perceived stress scale (PSS)

The perceived stress scale (PSS) is a 14-item self-report questionnaire designed to measure tension and control ability under stress (Cohen et al., 1983). For each item, participants rated their degree of stress using Likert 5-level scoring. A total score greater than 28 indicates high-level stress. The Cronbach’s α of the PSS in the study was good (pre-/post-test) = 0.87/0.84.

#### 2.2.2. Student Life Stress Inventory (SLSI)

The Student Life Stress Inventory (SLSI) is a self-report questionnaire that measures the different types of stressors (frustrations, conflicts, pressures, changes, and self-imposed stressors) and the reactions to the stressors (physiological, emotional, behavioral, and cognitive) (Culhane et al., 2001; Sabih et al., 2013; Liu et al., 2020). The total score of SLSI was used in the present study. In this study, the Cronbach’s α is 0.88 (pre-test) and 0.87 (post-test).

#### 2.2.3. State-Trait Anxiety Inventory (STAI)

The State-Trait Anxiety Inventory (STAI) is an assessment tool used to measure and evaluate levels of state and trait anxiety through self-reporting (Knight et al., 1983). The questionnaire contains 40 items, of which 20 are used to determine the state anxiety (SA) levels and the other 20 to measure trait anxiety (TA). The Likert scale employed here ranges from 1 (almost never) to 4 (almost always). Higher scores indicate more intense anxiety. The Cronbach’s α for SA was 0.89 for the pre-test and 0.85 for the post-test, and the Cronbach’s α for TA was 0.83 for the pre-test and 0.73 for the post-test.

#### 2.2.4. Beck Depression Inventory (BDI)

The Beck Depression Inventory is a 21-item self-assessment tool used to evaluate the severity of depression (Beck et al., 1961). Responses are rated based on a scale of 0 to 3, which produces a score from 0 to 63. Scores higher than 14 generally signify that clinical depression is present. Higher scores indicate more severe depression. In our study, BDI had a Cronbach’s α (pre-/post-test) of 0.89/0.86.

#### 2.2.5. Positive and Negative Affect Schedule (PANAS)

The Positive and Negative Affect Schedule is a 20-item survey which allows individuals to evaluate their current emotional states (Watson et al., 1988). Participants evaluated 20 adjectives that depicted their current mood on a 5-point scale from 1 (very subtly or not at all) to 5 (extremely). The scores for positive and negative affect were summed up separately. The past studies that involved the positive affects schedule reported the Cronbach’s αs ranging from 0.86 to 0.90, and 0.84 to 0.87 for the negative affects schedule (Watson et al., 1988). In our study, the Cronbach’s α (pre-/post-test) of the positive affect was 0.80/0.83 and the Cronbach’s α (pre-/post-test) of the negative affect was 0.86/0.82.

### 2.3. Attention network test

**FIGURE 1:**
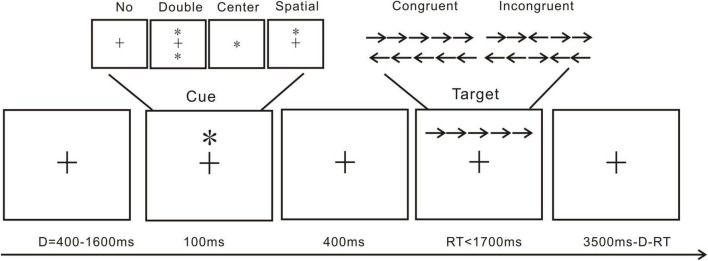
Trial from the attention network test: *, cue; →/←, arrow; +, fixation cross.

The attention network test (ANT; Figure 1) is conventionally used to explore attention alerting, attention orienting, and executive function (Fan et al., 2002). The present ANT referenced our previous investigation (Liu et al., 2020). Cue and target are included in the present ANT trials. As shown in Figure 1, a standard trial includes a cue and a target presentation. The cue presentation includes a fixation cross followed by double cue condition, in which the two cues would appear vertically above and below the fixation cross; center cue condition, meaning the cue would be presented at the center of the screen, replacing the fixation cross; and spatial cue conditions, in which one cue would appear vertically directly above the fixation cross. In the target presentation, the center arrow would be flanked by two arrows from each side. In a single trial, a fixation cross would first appear for 400–1,600 ms, then a 100 ms cue presentation, followed by another fixation cross presentation for 400 ms. Lastly, the targets would be presented at a maximum of 1,700 ms or till the participants made a response. All arrows pointing in the same direction constitute a congruent trial, and the vice versa makes an incongruent trial. The participants were asked to react to the center arrow direction. If the arrow was pointing to the left, the participants were instructed to press “F” with their left hand, and if it was pointing to the right, they were instructed to press “J.” Each trial lasted for a total of 4,000 ms. One practice block of 20 trials and one test block of 572 trials made up the ANT. For each cue type, approximately 140 trials appeared. For each target type, approximately 280 trials appeared. To minimize experimental artifacts in the EEG data, participants were instructed to maintain as much stillness as possible and to minimize eye blinks. Each participant‘s response time and accuracy for each condition were calculated after excluding trials with a response time of less than 200 ms and extreme data with over 3 standard deviations.

### 2.4. EEG recording and preprocessing

**FIGURE 2:**
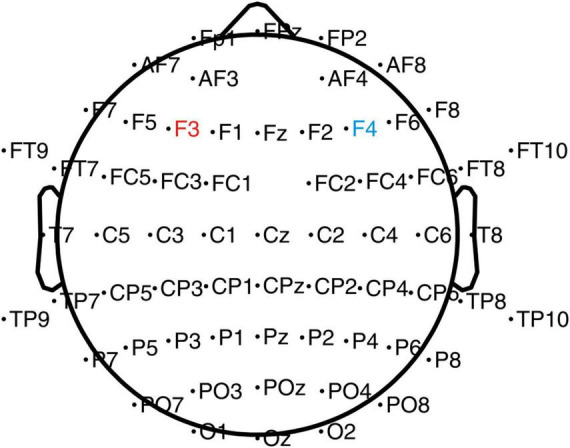
EEG and tDCS electrodes placement chart. The anode electrode (4 cm × 4 cm) was placed on the left DLPFC (F3), and the cathode electrode was placed on the right DLPFC (F4).

An elastic cap of 64 sites attached with tin electrodes (Brain Products GmbH, Gilching, Germany) was used to record the brain‘s electrical activity (see Figure 2). The reference electrode was placed on the FCz (fronto-central aspect) site, and the ground electrode was on the AFz (the medial frontal aspect) site. An electrode was placed infra-orbitally under the right eye for the vertical electrooculogram (IO). Throughout the fitting of the cap and the recording of the EEG, all inter-electrode impedance was kept below 5 KΩ.

The EEG data were preprocessed offline on EEGLAB (Delorme and Makeig, 2004), a toolbox from MATLAB that is mainly used for processing EEG data. Each ERP and the grand averages during the ANT were calculated. The data was first downsampled from 1,000 to 500 Hz (Liu et al., 2019). The EEG data were first digitally filtered with a 0.1–45 Hz bandpass filter and re-referenced to the average of the two mastoids. Epochs were established in relation to when the cue and target displays first appeared. Epochs were disregarded because their amplitude differences were greater than 100 V. Independent component analysis was used to find and remove muscle artifacts, eye movements, and blinks. Based on the previous studies, data were epoched from 200 ms before the start of the cues to 500 ms after the presentation, and from 200 ms before the start of the targets to 1,000 ms after the presentation (Liu et al., 2019, 2020).

### 2.5. tDCS parameters

A Low-Intensity transcranial DC Stimulator (Soterix Medical, Woodbridge, NJ, USA) was used in the present study. Based on the international EEG 10–20 system and previous studies (Austin et al., 2016; Dubreuil-Vall et al., 2019, 2021), stimulations were performed using 4 cm × 4 cm (16 cm^2^) electrodes (Figure 2) with the anode placed on the left DLPFC (F3) and cathode on the right DLPFC (F4). A previous study has shown that left-sided stimulation could potentially have a better effect than the right side (Dubreuil-Vall et al., 2021). During the stimulation, one investigator was aware of group randomization for the participants and was responsible for setting up the stimulator according to the protocol for the sham and the anodal-tDCS. The investigator was not involved in any other data collection process. The participants in the anodal tDCS group were stimulated with 2-mA for 20 min every day for 5 days. The ramp-up and down were set at the beginning and end of the stimulation for 30 s. In the sham tDCS group, to simulate the potential experience of local tingling sensation that real stimulation produces but without any sustained effects on the cortical activity, the electrodes were placed at the same locations as the anodal tDCS group, but the currents were only applied during the 30 s ramp-up phase at the beginning and ramp down phase at the end of the 20-min sham-stimulation period.

### 2.6. Procedure

**FIGURE 3:**
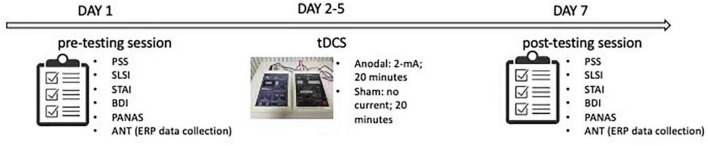
Experimental procedure. Perceived stress scale (PSS); Student life stress inventory (SLSI); State anxiety inventory (SAI); Trait anxiety inventory (TAI); Beck depression inventory (BDI); Positive and negative affect scale (PANAS); Attention network test (ANT).

As shown in Figure 3, the data collection process took 7 days to complete. On the first day, participants completed the pre-test of the PSS, the STAI, the BDI, the PANAS, and the ANT. For the next 5 days, each participant in the anodal tDCS group then received the stimulation at 2-mA for 20 min every day, while the participants in the sham group received the sham stimulation for 20 min every day. On the seventh day, all participants completed the post-test of the PSS, the STAI, the BDI, the PANAS, and the ANT.

### 2.7. Statistical analyses

All analyses were performed on the SPSS 22.0 software. The Greenhouse–Geisser method was used to adjust for sphericity. Bonferroni *post-hoc t*-tests were conducted for the multiple comparisons. An α level below 0.05 was considered statistically significant for each analysis. The analyses were based on the means from the 283 trials for the target condition and 143 trials for the cue condition.

#### 2.7.1. Behavioral analyses

For each participant, the PSS, the STAI, the BDI, and the PANAS scores were calculated separately by summing up the responses. The summed up score of each tests were used to investigate the difference in depression and stress before and after the stimulation between the two groups. Four 2 (group: anodal and sham tDCS group) × 2 (test: pre- and post-test) ANOVAs were carried out. The group was set as a between-subjects factor, and the test was set as a within-subjects factor.

To compare the performance of the ANT in the two groups, two 2 (group: anodal and sham tDCS group) × 2 (test: pre- and post-test) × 2 (target: congruent and incongruent) ANOVAs were conducted for the reaction time and accuracy. Each participant’s response time and accuracy for each condition were calculated after excluding trials with a response time of less than 200 ms and extreme data with over 3 standard deviations. The group was the between-subjects factor, test and target were the within-subject factors in the analysis.

In addition, three 2 (group: anodal and sham group) × 2 (test: pre- and post-test) ANOVAs were conducted for alerting effect, orienting effect, and executive control effect, with the group as a between-subjects factor, and the test as a within-subjects factor. The altering effect was computed by deducting the mean reaction time of the double-cue condition from the mean reaction time of the no-cue condition. The orienting effect was computed by deducting the mean reaction time of the spatial cue condition from the mean reaction time of the center cue. The executive control effect was computed by deducting the mean reaction time of all congruent target conditions from that of the incongruent target conditions (Fan et al., 2002). A greater value of alerting and orienting effect indicates better alerting and orienting abilities, while a greater value of the executive control effect indicates worse executive control ability.

#### 2.7.2. EEG analyses

On the basis of the topographical distribution of the grand-averaged ERP activities, the N1, P2, N2, and P3 potentials, and Fz, FCz, Cz, CPz, and Pz sites were evaluated. The followings are the ERP’s component time epochs and their components: N1, 100–150 ms; P2, 150–200 ms; N2, 200–300 ms; and P3, 300–450 ms. Four 2 (group: anodal/sham group) × 2 (test: pre-/post-test) × 4 (cue: no/center/double/spatial) × 5 (sites: Fz/FCz/Cz/CPz/Pz) ANOVAs were conducted, in which the group was a between-subjects factor, test, cue, and sites were the within-subject factors.

For investigating the ERP difference in reaction to different targets, the potentials and their time windows selected were as follows: P2, 150 –200 ms; N2, 200 –330 ms; P3, 330 –480 ms. Three 2 (group: anodal/sham group) × 2 (test: pre-/post-test) × 2 (target: congruent/incongruent) × 5 (sites: Fz/FCz/Cz/CPz/Pz) ANOVAs were conducted with group as a between-subjects factor, test, target, and sites as the within-subject factors. According to the outlier analyses on the EEG data using ± 3 SDs, all EEG data were within range. Therefore, no EEG data were discarded.

## 3. Results

### 3.1. Self-report results

**FIGURE 4:**
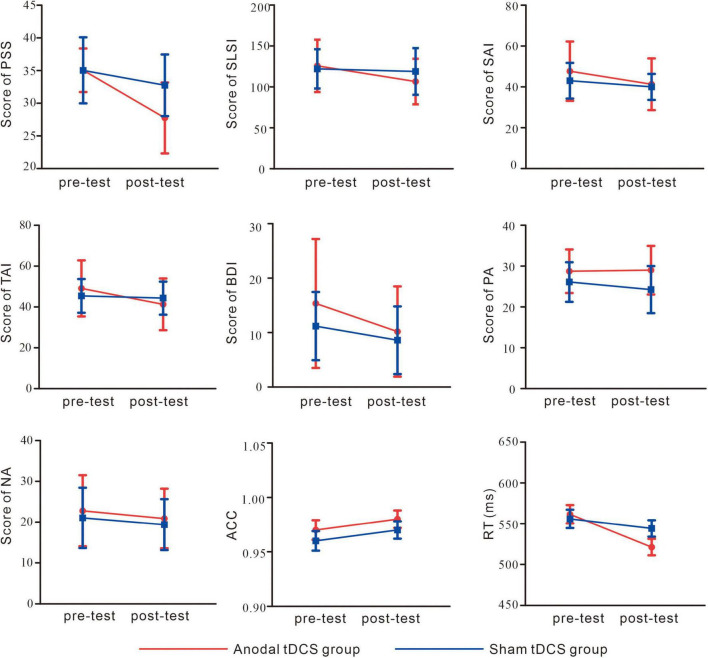
Self-report and behavioral results. perceived stress scale (PSS); Student Life Stress Inventory (SLSI); State Anxiety Inventory (SAI); Trait Anxiety Inventory (TAI); Beck Depression Inventory (BDI); Positive Affect (PA); Negative Affect (NA); accuracy (ACC); reaction time (RT).

As shown in Figure 4 and Table 1, for PSS, an interaction between group and test was found, *F*(1,38) = 8.93, *p* = 0.01, partial η*^2^* = 0.19. While no significant difference between the groups in the pre-test of the PSS scores was found, *p* = 0.99, the anodal tDCS group exhibited a significantly lower post-test PSS score than the sham group, *p* = 0.004. The simple effect analysis showed that the PSS scores of the anodal tDCS group decreased significantly after the stimulation, *p* < 0.001. No similar effect was observed in the sham group, *p* = 0.06. In addition, The results also showed a main effect of the test, *F*(1,38) = 32.90, *p* < 0.001, partial η*^2^* = 0.46, such that the post-test PSS score was significantly lower than the pre-test scores, *p* < 0.001.

**TABLE 1:** Descriptive statistics for the background measures.

Variable	Anodal tDCS (*n* = 20)		Sham tDCS (*n* = 20)	
	Pre-test Mean (SD)	Post-test Mean (SD)	Pre-test Mean (SD)	Post-test Mean (SD)
PSS	35.05 (3.33)	27.75 (5.43)	35.04 (5.06)	32.75 (4.72)
SLSI	125.80 (32.09)	106.55 (27.83)	122.05 (23.98)	118.95 (28.47)
SAI	47.7 (14.52)	37.20 (11.11)	43.05 (8.73)	40.00 (6.35)
TAI	49.10 (13.76)	41.30 (12.67)	45.45 (8.28)	44.35 (8.09)
BDI	15.35 (11.83)	10.20 (8.29)	11.20 (6.25)	8.60 (6.22)
PA	28.75 (5.33)	29.00 (5.96)	26.10 (4.85)	24.25 (5.78)
NA	22.80 (8.72)	20.90 (7.33)	21.05 (7.41)	19.40 (6.24)
ACC	0.97 (0.009)	0.98 (0.008)	0.96 (0.009)	0.97 (0.008)
RT	561.61 (11.26)	521.32 (10.07)	555.93 (11.26)	544.23 (10.07)

PSS, perceived stress scale; SLSI, student-life stress inventory; SAI, state anxiety inventory; TAI, trait anxiety inventory; BDI, Beck Depression Inventory; PA, positive affect; NA, negative affect; RT, reaction time in ms; ACC, accuracy rate; SD, standard deviation.

As shown in Figure 4 and Table 1, the results on the SLSI scores showed an interaction between group and test, *F*(1,38) = 4.41, *p* = 0.04, partial η*^2^* = 0.10. The simple effect analysis revealed that the SLSI scores decreased significantly in the anodal tDCS group, *p* = 0.001, while no effect was observed in the sham group, *p* = 0.57. There was no significant difference between the anodal tDCS and the sham group in the pre-test, *p* = 0.68.

As shown in Figure 4 and Table 1, the results on the SAI scores revealed an interaction of group and test, *F*(1,38) = 11.05, *p* = 0.002, partial η*^2^* = 0.23. The simple effect analysis revealed that the SAI scores significantly decreased in the anodal tDCS group, *p* < 0.01, while no effect was observed in the sham group, *p* = 0.06. There was no significant difference between the groups in neither the pre- (*p* = 0.23) nor the post-test (*p* = 0.33). The *post-hoc* test exhibited a main effect of the test, *F*(1,38) = 36.57, *p* < 0.001, partial η*^2^* = 0.49, in which showed a significant decrease in the SAI score after the anodal stimulation, *p* < 0.001.

As shown in Figure 4 and Table 1, there was no significant difference between groups both in the pre- (*p* = 0.32) and post-test (*p* = 0.37). The simple effect analysis showed that the TAI scores significantly decreased after the anodal tDCS, *p* < 0.001, and not after the sham tDCS, *p* = 0.46. The *post-hoc* test also showed a main effect of the test, *F* (1, 38) = 18.32, *p* < 0.001, partial η*^2^* = 0.33, for which in the anodal tDCS group, the TAI scores were significantly lowered, *p* < 0.001.

We did not observe an interaction of group and test on the BDI scores, *F*(1,38) = 1.20, *p* = 0.28, partial η*^2^* = 0.03. However, a main effect of the test was found, *F*(1,38) = 11.11, *p* = 0.002, partial η*^2^* = 0.23, in the *post-hoc* test, the anodal group exhibited significantly lower BDI scores in the post-test, *p* = 0.002 (see Table 1).

No interaction between the group and test, *F*(1,38) = 2.20, *p* = 0.15, partial η*^2^* = 0.06, nor the main effect of the test, *F*(1,38) = 1.28, *p* = 0.27, partial η*^2^* = 0.03 on the PA scores. However, the results on the NA scores showed a main effect of the test, *F* (1, 38) = 4.91, *p* = 0.03, partial η*^2^* = 0.11, for which a *post-hoc* test showed significantly lowered NA scores in the post-test in the anodal tDCS group, *p* = 0.03 (see Table 1).

### 3.2. Behavioral results

The results of the reaction time (Figure 4) showed an interaction of group and test, *F*(1,38) = 5.86, *p* = 0.02, partial η*^2^* = 0.13. The simple effect analysis showed that the anodal tDCS group showed a significantly greater reaction time in the pre-test compared with that in the post-test, *p* < 0.001 while no significant difference was found in the sham group, *p* = 0.17. There were no differences found between the two groups in the pre- (*p* = 0.72) or post-test (*p* = 0.12). The results also showed a main effect of the test, *F*(1,38) = 19.37, *p* < 0.001, partial η*^2^* = 0.34. Reaction time in the pre-test was significantly greater compared with that in the post-test. The main effect of the target, *F*(1,38) = 4.43, *p* = 0.04, partial η*^2^* = 0.10, showed that for both groups, the reaction time in the incongruent trials was greater than that in the congruent trials.

The results on accuracy (Figure 4) did not show an interaction of group and test, *F*(1,38) = 0.07, *p* = 0.78, partial η*^2^* = 0.002, main effects of test, *F*(1,38) = 3.23, *p* = 0.08, partial η*^2^* = 0.078 or of the target, *F*(1,38) = 0.08, *p* = 0.93, partial η*^2^* < 0.001.

### 3.3. ERP results

#### 3.3.1. Cue-related ERPs

**FIGURE 5:**
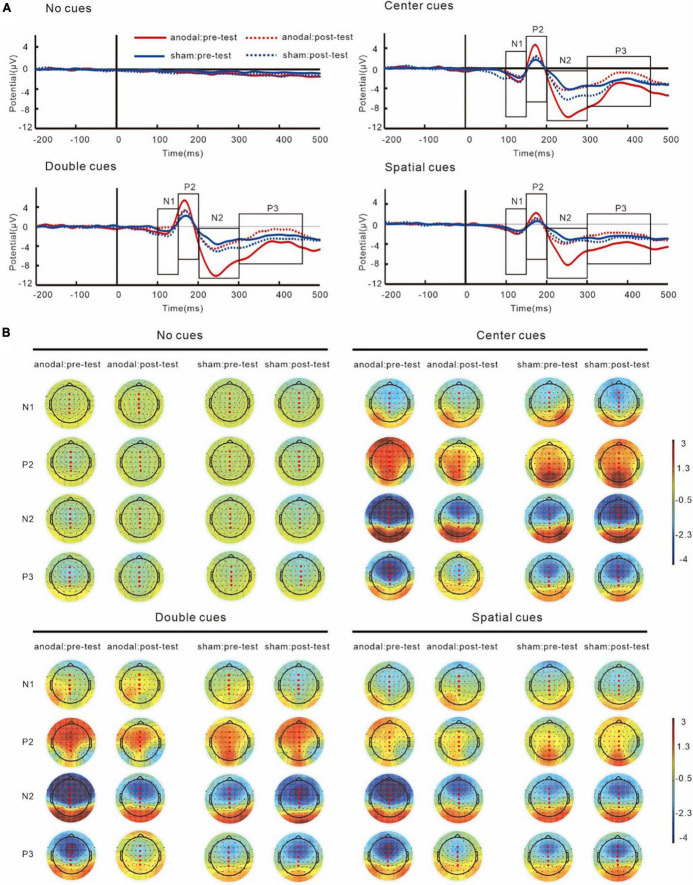
Grand average ERPs **(A)** and topography plots **(B)** of cue-N1, cue-P2, cue-N2, and cue-P3. Electrode selected for the analyses are highlighted in red.

Grand average ERPs and topography plots for cue-N1, cue-P2, cue-N2, and cue-P3 are shown in Figure 5 and Table 2. Results on N1 showed no interaction of group and test, *F*(1,38) = 3.86, *p* = 0.06, partial η*^2^* = 0.09. However, a main effect of cue was found, *F*(3,114) = 11.01, *p* < 0.001, partial η*^2^* = 0.23, which showed that the amplitudes of cue-N1 were greatest for center cues. The results also showed a main effect of site, *F*(4,152) = 9.25, *p* = 0.002, partial η*^2^* = 0.20, in which the amplitudes of cue-N1 were the greatest at Fz.

**TABLE 2:** The values of amplitudes of cue-N1, cue-P2, cue-N2, cue-P3, target-P2, target-N2, and target-P3.

Variable	tDCS group (*n* = 20; M ± SD)		Sham group (*n* = 20; M ± SD)	
	Pre-test	Post-test	Pre-test	Post-test
cue-N1	−0.79 (0.32)	−0.47 (0.25)	−0.53 (0.32)	−1.14 (0.25)
cue-P2	1.16 (0.29)	0.58 (0.39)	0.86 (0.29)	0.76 (0.39)
cue-N2	−3.71 (0.58)	−1.81 (0.49)	−1.36 (0.58)	−2.22 (0.50)
cue-P3	−2.46 (0.49)	−0.94 (0.36)	−1.24 (0.49)	−1.77 (0.36)
target-P2	1.18 (0.24)	0.96 (0.26)	−0.18 (0.24)	−0.42 (0.26)
target-N2	−3.43 (0.43)	−1.99 (0.47)	−1.70 (0.43)	−2.29 (0.47)
target-P3	−2.04 (0.31)	−1.04 (0.31)	−1.29 (0.31)	−1.56 (0.31)

Similar with N1, analyses on P2 did not show any interactions of group and test, *F*(1,38) = 0.93, *p* = 0.34, partial η*^2^* = 0.02. However, the results did show a main effect of cue, *F*(3,114) = 31.34, *p* < 0.001, partial η*^2^* = 0.45, with greatest cue-P2 amplitudes to center cues.

For N2 results, an interaction between group and test was found, *F*(1,38) = 7.42, *p* = 0.01, partial η*^2^* = 0.16. The simple effect analysis revealed that post-test cue-N2 amplitudes decreased significantly compared to the pre-test in the anodal tDCS group, *p* = 0.01. However, there was no significant difference between the pre- and post-test in the sham group, *p* = 0.24. Pre-test cue-N2 amplitudes in the anodal tDCS group were greater compared with those in the sham group, *p* = 0.01, while there was no significant difference between the groups in the post-test, *p* = 0.57; The results also indicated a main effect of cue, *F*(3,114) = 33.56, *p* < 0.001, partial η*^2^* = 0.47, the amplitudes of N2 were greatest during double cues. The results also showed a main effect of sites, *F*(4,152) = 65.45, *p* < 0.001, partial η*^2^* = 0.63, with greatest cue-N2 amplitudes at site Fz.

Results on P3 also showed an interaction of group and test, *F*(1,38) = 7.15, *p* = 0.01, partial η*^2^* = 0.16. The simple effect analysis showed that in the anodal tDCS group, post-test P3 amplitudes increased significantly compared to the pre-test, *p* = 0.008. However, no significant difference was found between the pre- and post-test amplitudes in the sham group, *p* = 0.33. There was no between-group difference in the pre- (*p* = 0.09) or post-test (*p* = 0.11). The results also showed a main effect of sites, *F*(4,152) = 51.82, *p* < 0.001, partial η*^2^* = 0.58, in which the amplitudes of cue-P3 were the greatest at Pz.

#### 3.3.2. Target-related ERPs

**FIGURE 6:**
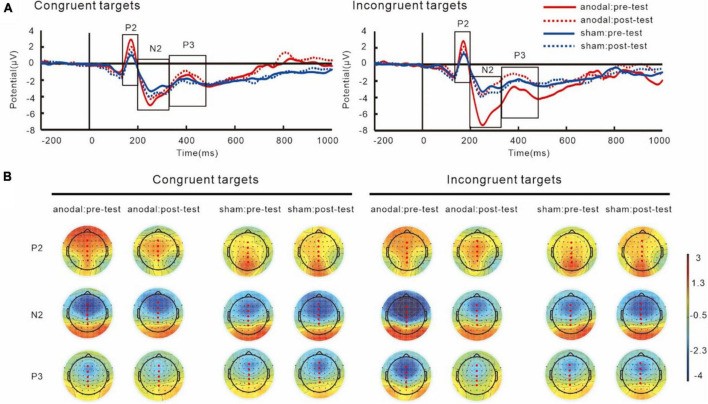
Grand average ERPs **(A)** and topography plots **(B)** for target-P2, target-N2, and target-P3. Electrode selected for the analyses are highlighted in red.

Grand averages of P2, N2, and P3 and their topography plots in reaction to different targets are shown in Figure 6 and Table 2.

For P2 components, the results showed an interaction of group and target, *F*(1,38) = 12.38, *p* = 0.001, partial η*^2^* = 0.25. The simple effect analysis showed that the target-P2 amplitudes was significantly greater in the anodal tDCS group than in the sham group, *p* < 0.001. The results also showed a main effect of target, *F*(1,38) = 12.18, *p* = 0.001, partial η*^2^* = 0.24, in both groups, the P2 amplitudes in the incongruent trials were greater than those in the congruent trials, *p* < 0.001. We did not find an interaction between group and test, *F*(1,38) = 0.001, *p* = 0.97, partial η*^2^* = 0.001.

The results on N2 amplitudes showed an interaction between group and test, *F*(1,38) = 6.61, *p* = 0.01, partial η*^2^* = 0.15. The simple effect analysis showed that in the anodal tDCS group, the post-test target-N2 amplitudes were significantly lower than the pre-test amplitudes, *p* = 0.01, while no difference between the pre- and post-tests amplitudes was observed in the sham group, *p* = 0.29. We also found that the pre-test N2 amplitudes in the anodal tDCS group were greater than the pre-test N2 amplitudes in the sham group, *p* = 0.01, whereas no significant group difference in the post-test was found, *p* = 0.65. The results also revealed a main effect of sites, *F*(4,152) = 82.19, *p* < *0*.001, partial η*^2^* = 0.68, with the greatest amplitudes at Fz.

Analyses on P3 also showed an interaction of group and test, *F*(1,38) = 5.83, *p* = *0*.02, partial η*2* = 0.13. The simple effect analysis showed that post-test target-P3 amplitudes significantly increased compared to the pre-test in the anodal tDCS group, *p* = 0.01 while no significant difference between pre- and post-tests in the sham group was found, *p* = 0.48. There were no differences between the groups in the pre- (*p* = 0.10) or post-test (*p* = 0.24). The results also showed a main effect of sites, *F*(4,152) = 81.01, *p* < *0*.001, partial η*^2^* = 0.68, where the amplitudes of target-P3 were greatest at Pz.

In addition, we found that there were significant differences between two groups in pre-test N2 amplitudes in reaction to both different cues and targets. Therefore, the changes (post-test minus pre-test) in cue-N2 and target-N2 between two groups were analyzed in the current study.

### 3.4. Relationship between the changes in ERPs and behavior indexes

We explored the relationship between changes in ERPs (pre-test minus post-test) and reaction time (pre-test minus post-test). As presented in Table 3, Pearson’s correlation analysis showed that a significant weak negative correlation between the changes in reaction time during incongruent trials and the changes in the target-P3 during the congruent trails (*r* = −0.32, *p* = 0.04) and cue-P3 (*r* = −0.34, *p* = 0.03). In addition, the changes in the PSS score were moderately negatively related to changes in target-P3 amplitudes during congruent trials (*r* = −0.45, *p* = 0.003).

**TABLE 3:** Relationship between the changes in ERPs and behavior indexes.

Variable	Cong-RT	Incong-RT	PSS Score
T cong-P2	−0.17 (0.28)	−0.17 (0.29)	−0.03 (0.88)
T incong-P2	0.06 (0.71)	0.03 (0.88)	0.05 (0.77)
T cong-N2	−0.16 (0.33)	−0.14 (0.41)	0.11 (0.50)
T incong-N2	−0.15 (0.35)	−0.15 (0.34)	−0.05 (0.77)
T cong-P3	−0.30 (0.06)	−0.32* (0.04)	−0.45** (0.003)
T incong-P3	−0.13 (0.41)	−0.13 (0.43)	−0.28 (0.08)
Cue-N1	−0.11 (0.49)	−0.10 (0.53)	−0.01 (0.97)
Cue-P2	0.01 (0.94)	−0.23 (0.15)	−0.06 (0.72)
Cue-N2	−0.22 (0.18)	−0.004 (0.98)	0.14 (0.38)
Cue-P3	−0.28 (0.08)	−0.34* (0.03)	−0.29 (0.07)

Cong-RT, reaction time during the congruent target trials; Incong-RT, reaction time during the incongruent target trials; T cong-P2, target-P2 during congruent trials; T incong-P2, target-P2 during incongruent trials; T cong-N2, target-N2 during congruent trials; T incong-N2, target-N2 during incongruent trials; T cong-P3, target-P3 during congruent trials; T incong-P3, target-P3 during incongruent trials. *p < 0.05, **p < 0.01.

## 4. Discussion

In our study, we investigated the effects of tDCS on attentional control in chronically stressed individuals. Based on our present results, tDCS targeting the left DLPFC could relieve chronic stress, which was confirmed by the decreased perceived stress and anxiety in the post-test of the anodal tDCS group compared with the pre-test and the sham tDCS modulation. After the anodal tDCS, participants showed more substantial attentional control than those in the sham group, which was reflected by the faster reaction time during ANT. Target-P2 amplitudes were significantly greater in the anodal tDCS group than those in the sham group. Importantly, we discovered that anodal tDCS significantly reduced N2 amplitudes (both cue- and target-N2). After the anodal tDCS, we also discovered a significant rise in P3 amplitudes (both cue- and target-P3). Furthermore, we discovered a correlation between the variations in cue- and target-P3 amplitudes and the variations in reaction times in incongruent trials. To the best of our knowledge, our study is the first to explore the effect of anodal tDCS administered to the left DLPFC using cognitive and physiological measures of attentional control in adults with chronic stress.

### 4.1. Anodal tDCS over the left DLPFC and attention control

According to earlier studies, the DLPFC is essential for processing negative emotions. According to Eippert et al.’s (2007) research, both up- and down-regulations of negative images activated the DLPFC). In a prior study using the Flanker task, healthy participants’ reaction times significantly improved after the anodal tDCS was administered to their left DLPFC. The enhanced Flanker task performance demonstrated that the tDCS modulated response inhibition, conflict monitoring, and selective attention (Dubreuil-Vall et al., 2019). Dubreuil-Vall et al. (2021) explored the effect of tDCS on cognitive control in ADHD patients by targeting the left and the right DLPFC and found that during the Flanker task, the anodal tDCS to the left DLPFC modulated cognitive function, which was reflected by faster responses. However, the stimulation on the right side did not show similar improvements. Therefore, the left DLPFC might be crucial for cognitive control and attention. Our results remain consistent with the previous findings. In the present study, the tDCS administered to the left DLPFC reduced the perceived degrees of stress and anxiety, consistent with Peña-Gómez et al.’s (2011) study. They believed that tDCS enhances cognitive control over emotional experiences (Peña-Gómez et al., 2011). Being consistent with previous findings, our study found a significant decrease in RT in the anodal tDCS group, which indicated more adequate attentional control. The present results further confirmed that anodal DCS targeting the left DLPFC can improve task performance in the ANT.

### 4.2. Indications of the improved attentional control via ERP components

P2 is an upward spike potential occurring at about 150–250 ms after the stimuli onset. It is associated with selective attention during stimulus evaluations (Luck et al., 1994; Potts, 2004; Gajewski et al., 2008). Enhanced P2 amplitude was typically considered to be related to how much attention was allocated to certain tasks (Nowicka et al., 2009). Individuals with ADHD showed reduced P2 amplitudes in the attention selection task compared to healthy controls (Johnstone et al., 2009). In the present study, target P2 amplitudes were significantly higher in the tDCS-anode group than in the sham group, suggesting that those in the tDCS-anode group allocated more attentional resources to targets, their tasks at hand, than the controls.

N1 is also used to explore the attentional processes and is typically related to selective attention (Xu et al., 2006). N1 are responsive to stimulus type. More precisely, N1 amplitudes are greater for stimuli that are processed than those that are ignored (Eason et al., 1969). Therefore, N1 reflects the correct allocation of attentional resources and the operation of the essential attentional sensory guarding and protection mechanism (Wang et al., 2019). Given that P2 and N1 amplitudes did not change after the anodal tDCS in the present study, we cautiously claimed that the tDCS administered to left DLPFC may have no significant effect on early attentional processing in chronically stressed adults.

N2 is a downward spike potential occurring at about 200–350 ms after the stimuli onset. N2 is closely linked with attentional processing, specifically, it is related to the recognition of novelty and mismatch; cognitive control, which includes response inhibition and conflict; and lastly, visual attention. According to earlier studies, N2 can reflect the effectiveness of the detection and monitoring of response conflicts when asked to make an incorrect or correct response (Folstein and Van Petten, 2008). The conflict-monitoring model asserts that during flanker tasks, larger N2 amplitudes would suggest that participants paid more attention to task-irrelevant than task-relevant information (Danielmeier et al., 2009). In addition, from the signal detection theory’s perspective, larger N2 amplitudes also reflect greater attentiveness and effort. According to the theory, the reduction of N2 amplitudes would mean a better signal-to-noise ratio, which is the ratio of useful information to useless ones, and more effortless and adequate use of the available cognitive resources for stimuli detection (Dubreuil-Vall et al., 2019). In our study, we found a reduction in N2 amplitudes (both cue- and target-N2 amplitudes) after the anodal tDCS. Our results were in line with a prior study, which revealed a decrease in N2 amplitudes in a Flanker task after the anodal tDCS over the left DLPFC brain region. They explained the decrease in N2 amplitudes following the anodal tDCS as a reduction in flanker distractions brought on by the improved selective attention capacity and decreased effort required to complete the task (Danielmeier et al., 2009). Therefore, taking into account current theories and prior research, the decrease in N2 amplitudes following stimulation discovered in the present study may point to an improvement in attentional control during the ANT.

P3 is an upward spiking potential present at about 300–600 ms after the stimuli onset and is closely related to the magnitude of attentional processing (Polich and Kok, 1995; Albert et al., 2013). Greater P3 amplitudes are related to enhanced post-conflict processing ability and subsequent inhibition of incorrect responses (Enriquez-Geppert et al., 2010; Clayson and Larson, 2011). Our study results revealed a negative mean P3 amplitude in both the pre and post-test of the sham and the anodal tDCS. Previous studies have shown that while acute stressors elicit an increased P3 amplitude, due to heightened attention and vigilance, chronic stress can lead to a reduction in P3 amplitude, reflecting cognitive impairments and attentional deficits (Liu et al., 2020). Previous studies have shown a reduction in target-P3 amplitudes in individuals with chronic stress, and they believed that individuals with chronic stress showed abnormal conflict control and impaired resource allocation abilities [(Liu et al., 2020) 26]. The reduction of P3 amplitudes was also found in individuals with attention deficits when compared to healthy individuals (Szuromi et al., 2011). In addition, Dubreuil-Vall et al. (2019) found enhanced P3 amplitudes after the anodal tDCSs in healthy people as well as ADHD patients and interpreted the increase in P3 amplitudes as an improvement and better modulation in conflict resolution, post-conflict resolution, and interference control, which subsequently led to an effective enhancement on distraction inhibition (Dubreuil-Vall et al., 2021). The present study also found an increase in P3 amplitudes in both cue- and target trials after the anodal tDCS. In contrast, no similar results were found in the controls, suggesting that the stimulation improved response inhibition and attentional control.

### 4.3. Relationship between the behavior indexes and the ERPs indexes

Our investigation revealed link between the changes in ERP indexes and the changes in the reaction times after the anodal tDCS. This would provide dual evidence, both behavioral and neural, for the effects of tDCS on the performance of the ANT. A previous finding indicated that the N2 and P3 potentials are related to conflict adaptation during the flanker tasks. The N2 amplitudes were positively related to reaction time in incongruent trials and P3 amplitudes were positively correlated with reaction time (Clayson and Larson, 2011). This would indicate that in incongruent trials, where more cognitive efforts were required and more conflicting information were being processed, higher N2 amplitudes would be displayed. At the same time, the P3 amplitudes follow the same logic. In our investigation, whereas the changes in P3 amplitudes were adversely associated to the changes in reaction time in the incongruent trials. This would indicate that while more cognitive resources being utilized in the incongruent trials, participants were able to process the conflicting information faster, hence the decreased reaction time. For congruent trials, there is a negative link between the changes in the P3 amplitudes and the PSS scores. This indicated as the more improvements exhibited in the PSS scores, the less the changes in the P3 amplitudes. In cases where the stimuli do not display conflicting information, less attentional efforts were required to process the stimuli. Our results would indicate that with improves stress scores, less attentional recourses were allocated to elicit a response, shown by the negative changes in the P3 amplitudes.

### 4.4. Limitations and future directions

To the best of our knowledge, our study is the first to investigate the attention-related neural markers of anodal tDCS targeting the left DLPFC in chronically stressed individuals. However, the limitations of the study should be knowledged. First, the sample size could have been bigger. The present ANT results were accompanied by a large percentage of accurate responses, leaving fewer incorrect trials to explore any significant changes in ERP amplitudes after the anodal tDCS. Future studies should recruit larger samples, use more challenging cognitive tasks to generate more incorrect trials, and further explore the effect of anodal tDCS. Second, the study lacked an active control group. Whether the same effect could be found by stimulating the right DLPFC (or other brain regions) should be further addressed in future studies with an active control group. Third, a follow-up study would be an ideal addition to the present results. We stay unclear on how long the anodal tDCS effect could last. In addition, we did not know whether the anodal tDCS could help with their postgraduate entrance examination performance. Future studies could include a longitudinal study.

## 5. Conclusion

All in all, our study exhibited supporting evidence for potential benefits of anodal tDCS for attention control in chronically stressed individuals. Specifically, the anodal tDCS targeting the left DLPFC brain region modulated participants’ moods, as shown by the reductions in perceived stress, state anxiety, and trait anxiety. In addition, the reduction in reaction time indicated an enhanced attentional control, which is also supported by the decrease in the N2 amplitudes and the increase in the P3 amplitudes.

## Data availability statement

The raw data supporting the conclusions of this article will be made available by the authors, without undue reservation.

## Ethics statement

The studies involving human participants were reviewed and approved by the Southwest University Ethics Committee. The patients/participants provided their written informed consent to participate in this study.

## Author contributions

YL and QL: conceptualization, methodology, and investigation. YL, QL, and JZ: formal analysis and visualization. QL, XL, and JH: data curation. HC: project administration and supervision. YL, FX, and HC: resources. YL and JZ: software. YL: writing—original draft preparation. YL, FX, YP, and HC: writing—review and editing. All authors have read and agreed to the published version of the manuscript.

## References

[B1] Albert J. López-Martín S. Hinojosa J. A. Carretié L. (2013). Spatiotemporal characterization of response inhibition. *Neuroimage* 76 272–281. 10.1016/j.neuroimage.2013.03.011 23523776

[B2] American Psychological Association (2014). Summary report of journal operations, 2013. *Am. Psychol.* 68, 381–382. 10.1037/a0033035 23895604

[B3] Austin A. Jiga-Boy G. M. Rea S. Newstead S. A. Roderick S. Davis N. J. (2016). Prefrontal electrical stimulation in non-depressed reduces levels of reported negative affects from daily stressors. *Front. Psychol.* 7:315. 10.3389/fpsyg.2016.00315 26973591 PMC4777740

[B4] Bari A. Robbins T. W. (2013). Inhibition and impulsivity: Behavioral and neural basis of response control. *Prog. Neurobiol.* 108 44–79. 10.1016/j.pneurobio.2013.06.005 23856628

[B5] Beck A. T. Ward C. H. Mendelson M. Mock J. Erbaugh J. (1961). An inventory for measuring depression. *Arch. Gen. Psychiatry* 4 561–571. 10.1001/archpsyc.1961.01710120031004 13688369

[B6] Boisseau C. L. Thompson-Brenner H. Caldwell-Harris C. Pratt E. Farchione T. Barlow D. H. (2012). Behavioral and cognitive impulsivity in obsessive-compulsive disorder and eating disorders. *Psychiatry Res.* 200 1062–1066. 10.1016/j.psychres.2012.06.010 22749228

[B7] Bradley M. M. Keil A. (2012). “Event-Related-Potentials,” in *Encyclopedia of human behavior*, 2nd Edn, ed. Ramachandran V. S. (Cambridge, MA: Academic Press), 79–85.

[B8] Carter C. S. van Veen V. (2007). Anterior cingulate cortex and conflict detection: An update of theory and data. *Cogn. Affect. Behav. Neurosci.* 7 367–379. 10.3758/cabn.7.4.367 18189010

[B9] Chen L. Oei T. P. Zhou R. (2023). The cognitive control mechanism of improving emotion regulation: A high-definition tDCS and ERP study. *J. Affect. Disord.* 332 19–28. 10.1016/j.jad.2023.03.059 36958485

[B10] Clayson P. E. Larson M. J. (2011). Effects of repetition priming on electrophysiological and behavioral indices of conflict adaptation and cognitive control. *Psychophysiology* 48 1621–1630. 10.1111/j.1469-8986.2011.01265.x 21806636

[B11] Coffman B. A. Trumbo M. C. Clark V. P. (2012). Enhancement of object detection with transcranial direct current stimulation is associated with increased attention. *BMC Neurosci.* 13:108. 10.1186/1471-2202-13-108 22963503 PMC3494452

[B12] Cohen S. Kamarck T. Mermelstein R. (1983). A global measure of perceived stress. *J. Health Soc. Behav.* 24 385–396. 6668417

[B13] Culhane J. F. Rauh V. McCollum K. F. Hogan V. K. Agnew K. Wadhwa P. D. (2001). Maternal stress is associated with bacterial vaginosis in human pregnancy. *Matern. Child Health J.* 5 127–134. 11573838 10.1023/a:1011305300690

[B14] Danielmeier C. Wessel J. R. Steinhauser M. Ullsperger M. (2009). Modulation of the error-related negativity by response conflict. *Psychophysiology* 46 1288–1298. 10.1111/j.1469-8986.2009.00860.x 19572907

[B15] David M. Moraes A. A. Costa M. L. D. Franco C. I. F. (2018). Transcranial direct current stimulation in the modulation of neuropathic pain: A systematic review. *Neurol. Res.* 40 555–563. 10.1080/01616412.2018.1453190 29600889

[B16] Delorme A. Makeig S. (2004). EEGLAB: An open source toolbox for analysis of single-trial EEG dynamics including independent component analysis. *J. Neurosci. Methods* 134 9–21. 15102499 10.1016/j.jneumeth.2003.10.009

[B17] Dubreuil-Vall L. Chau P. Ruffini G. Widge A. S. Camprodon J. A. (2019). tDCS to the left DLPFC modulates cognitive and physiological correlates of executive function in a state-dependent manner. *Brain Stimul.* 12 1456–1463. 10.1016/j.brs.2019.06.006 31221553 PMC6851462

[B18] Dubreuil-Vall L. Gomez-Bernal F. Villegas A. C. Cirillo P. Surman C. Ruffini G. (2021). Transcranial direct current stimulation to the left dorsolateral prefrontal cortex improves cognitive control in patients with attention-deficit/hyperactivity disorder: A randomized behavioral and neurophysiological study. *Biol. Psychiatry* 6 439–448. 10.1016/j.bpsc.2020.11.006 33549516 PMC8103824

[B19] Eason R. G. Harter M. R. White C. T. (1969). Effects of attention and arousal on visually evoked cortical potentials and reaction time in man. *Physiol. Behav.* 4 283–289.

[B20] Eippert F. Veit R. Weiskopf N. Erb M. Birbaumer N. Anders S. (2007). Regulation of emotional responses elicited by threat-related stimuli. *Hum. Brain Mapp.* 28 409–423. 10.1002/hbm.20291 17133391 PMC6871321

[B21] Enriquez-Geppert S. Konrad C. Pantev C. Huster R. J. (2010). Conflict and inhibition differentially affect the N200/P300 complex in a combined go/nogo and stop-signal task. *NeuroImage* 51 877–887. 10.1016/j.neuroimage.2010.02.043 20188191

[B22] Fan J. McCandliss B. D. Sommer T. Raz A. Posner M. I. (2002). Testing the efficiency and independence of attentional networks. *J. Cogn. Neurosci.* 14 340–347. 10.1162/089892902317361886 11970796

[B23] Folstein J. R. Van Petten C. (2008). Influence of cognitive control and mismatch on the N2 component of the ERP: A review. *Psychophysiology* 45 152–170. 10.1111/j.1469-8986.2007.00602.x 17850238 PMC2365910

[B24] Gajewski P. D. Stoerig P. Falkenstein M. (2008). ERP–correlates of response selection in a response conflict paradigm. *Brain Res.* 1189 127–134. 10.1016/j.brainres.2007.10.076 18053974

[B25] Gerrig R. J. (2007). *The psychology of survivor: Leading psychologists take an unauthorized look at the most elaborate psychological experiment ever conducted…Survivor!* Dallas, TX: Smart Pop.

[B26] Gonçalves ÓF. Rêgo G. Conde T. Leite J. Carvalho S. Lapenta O. M. (2018). Mind wandering and task-focused attention: ERP correlates. *Sci. Rep.* 8:7608. 10.1038/s41598-018-26028-w 29765144 PMC5953943

[B27] Hare T. A. Camerer C. F. Rangel A. (2009). Self-control in decision-making involves modulation of the vmPFC valuation system. *Science* 324 646–648. 10.1126/science.1168450 19407204

[B28] Haubert A. Walsh M. Boyd R. Morris M. Wiedbusch M. Krusmark M. (2018). Relationship of Event-related potentials to the vigilance decrement. *Front. Psychol.* 9:237. 10.3389/fpsyg.2018.00237 29559936 PMC5845631

[B29] Hillyard S. A. (2017). “Event-Related Potentials (ERPs) and cognitive processing,” in *Reference module in neuroscience and biobehavioral psychology*, ed. L. R. Squire (Amsterdam: Elsevier).

[B30] Jackowska M. Fuchs R. Klaperski S. (2018). The association of sleep disturbances with endocrine and perceived stress reactivity measures in male employees. *Br. J. Psychol.* 109 137–155. 10.1111/bjop.12250 28407210

[B31] Johnstone S. J. Barry R. J. Markovska V. Dimoska A. Clarke A. R. (2009). Response inhibition and interference control in children with AD/HD: A visual ERP investigation. *Int. J. Psychophysiol.* 72 145–153. 10.1016/j.ijpsycho.2008.11.007 19095016

[B32] Knight R. G. Waal-Manning H. J. Spears G. F. (1983). Some norms and reliability data for the State–Trait Anxiety Inventory and the Zung Self-Rating Depression scale. *Br. J. Clin. Psychol.* 22(Pt 4) 245–249. 10.1111/j.2044-8260.1983.tb00610.x 6640176

[B33] Koenen K. C. Ratanatharathorn A. Ng L. McLaughlin K. A. Bromet E. J. Stein D. J. (2017). Posttraumatic stress disorder in the World Mental Health Surveys. *Psychol. Med.* 47 2260–2274. 10.1017/s0033291717000708 28385165 PMC6034513

[B34] Lindfors P. Folkesson Hellstadius L. Östberg V. (2017). Perceived stress, recurrent pain, and aggregate salivary cortisol measures in mid-adolescent girls and boys. *Scand. J. Psychol.* 58 36–42. 10.1111/sjop.12347 28054380

[B35] Liu Q. Liu Y. Leng X. Han J. Xia F. Chen H. (2020). Impact of chronic stress on attention control: Evidence from behavioral and event-related potential analyses. *Neurosci. Bull.* 36 1395–1410. 10.1007/s12264-020-00549-9 32929635 PMC7674527

[B36] Liu Y. Zhao J. Zhang X. Gao X. Xu W. Chen H. (2019). Overweight adults are more impulsive than normal weight adults: Evidence from ERPs during a chocolate-related delayed discounting task. *Neuropsychologia* 133:8. 10.1016/j.neuropsychologia.2019.107181 31476320

[B37] Luck S. J. Hillyard S. A. Mouloua M. Woldorff M. G. Clark V. P. Hawkins H. L. (1994). Effects of spatial cuing on luminance detectability: Psychophysical and electrophysiological evidence for early selection. *J. Exp. Psychol.* 20 887–904. 10.1037/0096-1523.20.4.887 8083642

[B38] Lupien S. J. de Leon M. de Santi S. Convit A. Tarshish C. Nair N. P. (1998). Cortisol levels during human aging predict hippocampal atrophy and memory deficits. *Nat. Neurosci.* 1 69–73. 10.1038/271 10195112

[B39] Marks I. F. Nesse R. M. (1994). Fear and fitness: An evolutionary analysis of anxiety disorders. *Ethol. Sociobiol.* 15 247–261. 10.1016/0162-3095(94)90002-7

[B40] Martin D. M. Liu R. Alonzo A. Green M. Player M. J. Sachdev P. (2013). Can transcranial direct current stimulation enhance outcomes from cognitive training? A randomized controlled trial in healthy participants. *Int. J. Neuropsychopharmacol.* 16 1927–1936. 10.1017/s1461145713000539 23719048

[B41] McEwen B. S. Gianaros P. J. (2010). Central role of the brain in stress and adaptation: Links to socioeconomic status, health, and disease. *Ann. N. Y. Acad. Sci.* 1186 190–222. 10.1111/j.1749-6632.2009.05331.x 20201874 PMC2864527

[B42] Mendes A. J. Pacheco-Barrios K. Lema A. Gonçalves ÓF. Fregni F. Leite J. (2022). Modulation of the cognitive event-related potential P3 by transcranial direct current stimulation: Systematic review and meta-analysis. *Neurosci. Biobehav. Rev.* 132 894–907. 10.1016/j.neubiorev.2021.11.002 34742723

[B43] Miller E. K. Cohen J. D. (2001). An integrative theory of prefrontal cortex function. *Annu. Rev. Neurosci.* 24 167–202. 10.1146/annurev.neuro.24.1.167 11283309

[B44] Milner A. Aitken Z. Kavanagh A. LaMontagne A. D. Petrie D. (2017). Status inconsistency and mental health: A random effects and instrumental variables analysis using 14 annual waves of cohort data. *Soc. Sci. Med.* 189 129–137. 10.1016/j.socscimed.2017.08.001 28800450

[B45] Moezzi S. Ghoshuni M. Amiri M. (2021). Transcranial direct current stimulation (tDCS) effects on attention enhancement: A preliminary event related potential (ERP) study. *Curr. Psychol.* 42 8798–8804. 10.1007/s12144-021-02190-9

[B46] Nejati V. Heyrani R. Nitsche M. (2022). Attention bias modification through transcranial direct current stimulation (tDCS): A review. *Neurophysiol. Clin.* 52 341–353. 10.1016/j.neucli.2022.09.002 36241563

[B47] Nelson J. T. McKinley R. A. Golob E. J. Warm J. S. Parasuraman R. (2014). Enhancing vigilance in operators with prefrontal cortex transcranial direct current stimulation (tDCS). *Neuroimage* 85(Pt 3) 909–917. 10.1016/j.neuroimage.2012.11.061 23235272

[B48] Nishida K. Morishima Y. Pascual-Marqui R. D. Minami S. Yamane T. Michikura M. (2021). Mindfulness augmentation for anxiety through concurrent use of transcranial direct current stimulation: A randomized double-blind study. *Sci. Rep.* 11:22734. 10.1038/s41598-021-02177-3 34815458 PMC8610980

[B49] Nowicka A. Jednoróg K. Wypych M. Marchewka A. (2009). Reversed old/new effect for intentionally forgotten words: An ERP study of directed forgetting. *Int. J. Psychophysiol.* 71 97–102. 10.1016/j.ijpsycho.2008.06.009 18682264

[B50] Patel S. H. Azzam P. N. (2005). Characterization of N200 and P300: Selected studies of the Event-Related Potential. *Int. J. Med. Sci.* 2 147–154. 10.7150/ijms.2.147 16239953 PMC1252727

[B51] Peña-Gómez C. Vidal-Piñeiro D. Clemente I. C. Pascual-Leone Á Bartrés-Faz D. (2011). Down-regulation of negative emotional processing by transcranial direct current stimulation: Effects of personality characteristics. *PLoS One* 6:e22812. 10.1371/journal.pone.0022812 21829522 PMC3146508

[B52] Polich J. (2007). Updating P300: An integrative theory of P3a and P3b. *Clin. Neurophysiol.* 118 2128–2148. 10.1016/j.clinph.2007.04.019 17573239 PMC2715154

[B53] Polich J. Kok A. (1995). Cognitive and biological determinants of P300: An integrative review. *Biol. Psychol.* 41 103–146. 10.1016/0301-0511(95)05130-9 8534788

[B54] Potts G. F. (2004). An ERP index of task relevance evaluation of visual stimuli. *Brain Cogn.* 56 5–13. 10.1016/j.bandc.2004.03.006 15380870

[B55] Pruett S. B. (2003). Stress and the immune system. *Pathophysiology* 9 133–153. 10.1016/s0928-4680(03)00003-8 14567930

[B56] Railo H. Koivisto M. Revonsuo A. (2011). Tracking the processes behind conscious perception: A review of event-related potential correlates of visual consciousness. *Conscious. Cogn.* 20 972–983. 10.1016/j.concog.2011.03.019 21482150

[B57] Rêgo G. G. Gonçalves Ó. F. Boggio P. S. (2022). Attention neuroenhancement through tDCS or neurofeedback: A randomized, single-blind, controlled trial. *Sci. Rep.* 12:17613. 10.1038/s41598-022-22245-6 36266396 PMC9584934

[B58] Richard Clark C. Veltmeyer M. D. Hamilton R. J. Simms E. Paul R. Hermens D. (2004). Spontaneous alpha peak frequency predicts working memory performance across the age span. *Int. J. Psychophysiol.* 53 1–9. 10.1016/j.ijpsycho.2003.12.011 15172130

[B59] Sabih F. Siddiqui F. R. Baber M. N. (2013). Assessment of stress among physiotherapy students at Riphah Centre of Rehabilitation Sciences. *J. Pak. Med. Assoc.* 63 346–349. 23914635

[B60] Sarkis R. A. Kaur N. Camprodon J. A. (2014). Transcranial Direct Current Stimulation (tDCS): Modulation of Executive Function in Health and Disease. *Curr. Behav. Neurosci. Rep.* 1 74–85. 10.1007/s40473-014-0009-y

[B61] Saunders N. Downham R. Turman B. Kropotov J. Clark R. Yumash R. (2015). Working memory training with tDCS improves behavioral and neurophysiological symptoms in pilot group with post-traumatic stress disorder (PTSD) and with poor working memory. *Neurocase* 21 271–278. 10.1080/13554794.2014.890727 24579831

[B62] Shipstead Z. Harrison T. L. Engle R. W. (2015). Working memory capacity and the scope and control of attention. *Atten. Percept. Psychophys.* 77 1863–1880. 10.3758/s13414-015-0899-0 25911154

[B63] St Jacques P. L. Kragel P. A. Rubin D. C. (2013). Neural networks supporting autobiographical memory retrieval in posttraumatic stress disorder. *Cogn. Affect. Behav. Neurosci.* 13 554–566. 10.3758/s13415-013-0157-7 23483523 PMC3720739

[B64] Stoney C. M. Bausserman L. Niaura R. Marcus B. Flynn M. (1999). Lipid reactivity to stress: II, Biological and behavioral influences. *Health Psychol.* 18 251–261. 10.1037//0278-6133.18.3.251 10357506

[B65] Szuromi B. Czobor P. Komlósi S. Bitter I. (2011). P300 deficits in adults with attention deficit hyperactivity disorder: A meta-analysis. *Psychol. Med.* 41 1529–1538. 10.1017/S0033291710001996 20961477

[B66] van Rooij S. J. Geuze E. Kennis M. Rademaker A. R. Vink M. (2015). Neural correlates of inhibition and contextual cue processing related to treatment response in PTSD. *Neuropsychopharmacology* 40 667–675. 10.1038/npp.2014.220 25154707 PMC4289955

[B67] Wang X. Liu Y. She Y. Gao X. (2019). Neural correlates of appearance-based social comparison: The modulating effects of body dissatisfaction and person perspective. *Biol. Psychol.* 144 74–84. 10.1016/j.biopsycho.2019.03.007 30946871

[B68] Watson D. Clark L. A. Tellegen A. (1988). Development and validation of brief measures of positive and negative affect: The PANAS scales. *J. Pers. Soc. Psychol.* 54 1063–1070. 10.1037//0022-3514.54.6.1063 3397865

[B69] Wieser M. J. Keil A. (2020). Attentional threat biases and their role in anxiety: A neurophysiological perspective. *Int. J. Psychophysiol.* 153 148–158. 10.1016/j.ijpsycho.2020.05.004 32428525

[B70] Williams R. S. Biel A. L. Wegier P. Lapp L. K. Dyson B. J. Spaniol J. (2016). Age differences in the Attention Network Test: Evidence from behavior and event-related potentials. *Brain Cogn.* 102 65–79. 10.1016/j.bandc.2015.12.007 26760449

[B71] Xu G. Zhang Y. Hou H. Yan W. (2006). Event-related potential studies of attention to shape under different stimuli tasks. *Conf. Proc. IEEE Eng. Med. Biol. Soc.* Suppl., 6618–6621. 10.1109/iembs.2006.260902 17959467

[B72] Zhang X. Dong Y. Zhou R. (2018). Examination stress results in attentional bias and altered neural reactivity in test-anxious individuals. *Neural Plasticity* 2018:3281040. 10.1155/2018/3281040 29755511 PMC5884033

