# Corrigendum: Anodal transcranial direct current stimulation (tDCS) over the left dorsolateral prefrontal cortex improves attentional control in chronically stressed adults

**DOI:** 10.3389/fnins.2023.1310092

**Published:** 2023-10-26

**Authors:** Yong Liu, Qingjin Liu, Jia Zhao, Xuechen Leng, Jinfeng Han, Feng Xia, Yazhi Pang, Hong Chen

**Affiliations:** ^1^Key Laboratory of Cognition and Personality (Ministry of Education), Southwest University, Chongqing, China; ^2^School of Psychology, Southwest University, Chongqing, China; ^3^The Clinical Hospital of Chengdu Brain Science Institute, School of Life Science and Technology, University of Electronic Science and Technology of China, Chengdu, China; ^4^MOE Key Lab for Neuroinformation, High-Field Magnetic Resonance Brain Imaging Key Laboratory of Sichuan Province, University of Electronic Science and Technology of China, Chengdu, China; ^5^Department of Hepatobiliary Surgery, Southwest Hospital, Army Medical University, Chongqing, China; ^6^Faculty of Psychology, Research Center of Psychology and Social Development, Southwest University, Chongqing, China

**Keywords:** chronic stress, tDCS, left DLPFC, attentional control, N2, P3

In the published article, there was an error in affiliation(s) [3,4,5,6]. Instead of “^3^Department of Hepatobiliary Surgery, Southwest Hospital, Army Medical University, Chongqing, China

^4^Faculty of Psychology, Research Center of Psychology and Social Development, Southwest University, Chongqing, China”, it should be

“^3^The Clinical Hospital of Chengdu Brain Science Institute, School of Life Science and Technology, University of Electronic Science and Technology of China, Chengdu, China

^4^MOE Key Lab for Neuroinformation, High-Field Magnetic Resonance Brain Imaging Key Laboratory of Sichuan Province, University of Electronic Science and Technology of China, Chengdu, China

^5^Department of Hepatobiliary Surgery, Southwest Hospital, Army Medical University, Chongqing, China

^6^Faculty of Psychology, Research Center of Psychology and Social Development, Southwest University, Chongqing, China”.

The authors apologize for this error and state that this does not change the scientific conclusions of the article in any way. The original article has been updated.

